# OLDER ADULT PERSPECTIVES ON AGEISM DURING COVID-19: A QUALITATIVE STUDY

**DOI:** 10.1093/geroni/igac059.1258

**Published:** 2022-12-20

**Authors:** Carson De Fries, Pilar Ingle, Matthew Schilz, Andrew Steward, Emma Baker, Leslie Hasche

**Affiliations:** University of Denver, Denver, Colorado, United States; University of Denver, Lakewood, Colorado, United States; University of Denver, Denver, Colorado, United States; University of Wisconsin-Milwaukee, Milwaukee, Wisconsin, United States; University of Denver, Denver, Colorado, United States; University of Denver, Denver, Colorado, United States

## Abstract

Since the COVID-19 pandemic began, there has been a reported surge of ageism toward older adults. Research demonstrates that events perpetuating negative attitudes towards older adults can increase ageism and associated negative outcomes. The purpose of this phenomenological qualitative study was to explore how older adults navigated experiences of ageism and their social relationships during the COVID-19 pandemic. Semi-structured interviews with adults ages 60 and older were conducted between February and April of 2021 over Zoom. Data were coded using an iterative, inductive approach and thematic analysis was performed to draw themes from the data. A total of 24 participants ages 61-80 (mean = 70.6) were interviewed. Most participants identified as white (n = 19) female (n = 14), retired (n = 21) and had at least a bachelor’s degree (n = 22). Findings showed that participants experienced ongoing ageism but did not report ageist experiences associated specifically with COVID-19. Ageist experiences, unrelated to COVID-19, as shared by participants included assumptions about older adults’ (in)ability to use technology, ageism in professional settings, and feelings that ageism is an inevitable part of growing older. Future research should examine the impact of intersectionality on this topic within more diverse populations and explore potential differences that may have occurred throughout different stages of the pandemic.

